# The Effect of Intraoperative Transversus Abdominis Plane Blocking on Postoperative Pain After Laparoscopic Transabdominal Pre-peritoneal (TAPP) Groin Hernia Repair

**DOI:** 10.3389/fsurg.2022.834050

**Published:** 2022-02-08

**Authors:** Alexandros Chamzin, Maximos Frountzas, George Gkiokas, Evaggelia Kouskouni, Theodoros Xanthos, Nikolaos V. Michalopoulos

**Affiliations:** ^1^First Department of Propaeudeutic Surgery, Medical School, National and Kapodistrian University of Athens, Hippocration General Hospital, Athens, Greece; ^2^Department of Surgery, Medical School, National and Kapodistrian University of Athens, “Aretaieio” Hospital, Athens, Greece; ^3^Department of Biopathology, Medical School, National and Kapodistrian University of Athens, “Aretaieio” Hospital, Athens, Greece; ^4^Department of Health Sciences, School of Medicine, European University of Cyprus, Nicosia, Cyprus

**Keywords:** laparoscopy, groin hernia, analgesia, transversus abdominal plane, block

## Abstract

**Background:**

Managing postoperative pain even after laparoscopic groin hernia repair still remains an interesting challenge for clinicians especially for patients of high risk. Plenty of operative techniques and analgesic methods have been proposed in order to minimize postoperative pain after laparoscopic groin hernia repair. The aim of the present study is to compare transverse abdominis plane (TAP)-block with local analgesic infiltration at trocar entry sites in the terms of reducing postoperative pain.

**Methods:**

Patients that underwent laparoscopic trans-abdominal pre-peritoneal (TAPP) groin hernia repair in a high-volume university hospital were included. Patients were divided in two groups depending on the analgesic method used. Pain was assessed using Visual Numerical Scale (VNS) score.

**Results:**

Thirty patients were included. Intraoperative TAP-block seemed to be superior in terms of decreasing pain at the hernia area and at the trocar insertion site (*p* < 0.05) compared to local analgesic infiltration at the trocar insertion site at 6, 12 and 24 h after surgery (*p* < 0.05). In addition, pain reduction was more effective in rest rather than in motion for both analgesic methods.

**Conclusion:**

Intraoperative TAP-block under direct vision seems to be an effective, fast and easy technique in order to reduce postoperative pain after laparoscopic groin hernia repair, but more studies are required to validate these results in a prospective and randomized context.

## Introduction

Groin hernia repair was the most common surgical operation during 2018, as more than 20 million procedures had been performed worldwide (1). Laparoscopic repair of groin hernias has been emerged into a trend among surgical community due to multiple factors including faster postoperative recovery and return to daily routine, lower recurrence rate, reduced postoperative pain and excellent aesthetic results (2).

Laparoscopic technique in combination to regional infusion of local analgesic agents contribute in reducing postoperative pain after laparoscopic groin hernia repair (3, 4). However, it is relatively common that numerous patients complain about moderate pain near the trocar insertion sites, especially at the main 10 mm-umbilical port site, despite local anesthetic infiltration. Therefore, surgical community needs to pay more attention toward the management of postoperative pain after laparoscopic groin hernia repair in order to improve patient satisfaction (5).

Several studies have reported that laparoscopic repair of groin hernias combined with ultrasound (US)-guided transverse abdominis plane (TAP) blocking might have promising outcomes in alleviating postoperative pain (6, 7). During this technique, an injection of local anesthetic agents is performed under US-guidance between transverse and internal oblique muscle in order to infiltrate nerve fibers T7-L1, which innervate the anterior and lateral abdominal wall (8, 9). The purpose of this study is to investigate whether intraoperative TAP-block under direct vision could be an effective method of reducing postoperative pain after laparoscopic groin hernia repair.

## Materials and Methods

### Study Design and Participants

Patients that underwent laparoscopic trans-abdominal pre-peritoneal (TAPP) groin hernia repair with polypropylene mesh placement under general anesthesia at a university surgical department from January 2019 to April 2019 were included. Exclusion criteria were complicated hernias (large, irreducible, strangulated), immunosuppression, surgery within the last 3 months, chronic pain at the area, anticoagulation therapy, inability to answer or sign a consent form, cognitive disorders or refusal to sign a consent form and inability to undergo surgical procedures under general anesthesia.

Patients were retrospectively enrolled in our study. The study was performed according to the World Medical Association Declaration of Helsinki and ethical approval had been received by the institutional ethics committees. Our study was based on the evaluation of the clinical data of patients that were kept in record after informed consent obtainment, according to the institutions' policy.

### Surgical Procedures

**Figure 1:**
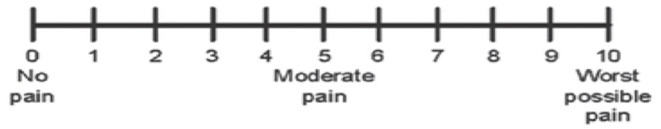
Visual Analog Scale score.

The study group consisted of patients that received intra-operative TAP-block under direct vision, whereas the control group consisted of patients that underwent local infiltration of anesthetic agent at the working trocars entry points. The aim of TAP-block was to deposit local anesthetic in the plane between the internal oblique and transversus abdominis muscles aiming the spinal nerves that pass along this plane. For TAP-block, 15 mL of 0.25% bupivacaine hydrochloride was used after laparoscopic procedures. In bilateral hernias, 10 mL of local anesthetic was injected in each side, due to the maximum dose allowed for each patient. The entry point of local anesthetic was the lumbar triangle of Petit, between the lower costal margin and iliac crest. It is bound anteriorly by the external oblique muscle and posteriorly by the latissimus dorsi muscle (10). For trocar entry points, 10 mL of 0.25% bupivacaine hydrochloride was used for incisions >8 mm and 5 ml of 0.25% bupivacaine hydrochloride was used for incisions <5 mm after laparoscopic procedures (11). Surgical and anesthesiology teams were the same for all patients. During the early postoperative period, all patients received 1 g paracetamol every 8 h and 8 mg lornoxicam 12 h after surgery. As rescue analgesia, 50 mg pethidine hydrochloride was administered intramuscularly. Postoperative pain at rest and during movement was assessed using the Visual Numerical Scale (VNS) score, ranging between 0–10, at the first 6, 12 and 24 h after surgery (Figure 1).

### Statistical Analysis

Power analysis was utilized to determine the minimum sufficient number of participants in order to achieve statistical significance. On this basis, a total sample of 30 patients (15 patients in each arm) was considered sufficient at bilateral levels of significance of 0.05. Mean and median values, standard deviations (SD) and interquartile ranges were used to describe quantitative variables. Absolute (N) and relative (%) frequencies were used to describe qualitative variables. Two-way repeated analysis (two-way repeated ANOVA) was used in order to investigate differences among the methods of anesthesia, infusion points and postoperative time intervals. Due to abnormality of data distribution, logarithmic transformations of the variables were performed. Analysis was performed using the SPSS version 22.0 software (SPSS Inc., Chicago, IL, USA). The statistical significance was set at a value of *p* < 0.05.

## Results

Thirty patients, 29 men (96.7%) and 1 woman (3.3%), were included in the present study. Inguinal hernia was present in 96.7% of patients, mainly on the right side (51.7%), whereas 3.3% of patients presented with femoral hernia on the left side. The demographic characteristics of included patients are presented in Table 1.

**Table 1:** Demographic characteristics of the study participants.

	**Control team (*n* = 15)**	**Intervention team (*n* = 15)**
Men	15	14
Women	0	1
Age, mean (SD)	58.8 (9.5)	49 (13.2)
Unilateral hernia	12	11
Bilateral hernia	3	4

### Pain at Rest

Pain scores at rest near the trocar insertion site and near the hernia area according to the 10-point VAS scale are presented in Table 2.

**Table 2:** Pain scores according to the 10-point VAS scale of patients at rest measured at the trocar and hernia site.

		**6 h**		**12 h**		**24 h**	
		**Mean (SD)**	**Median (range)**	**Mean (SD)**	**Median (range)**	**Mean (SD)**	**Median (range)**
Trocar placement site	Infiltration of trocar entry points	2.7 (0.7)	3 (2–3)	2.8 (0.7)	3 (2–3)	2.7 (0.7)	3 (2–3)
	Blockage of transverse abdominal muscle	0.5 (0.7)	0 (0–1)	0.5 (0.7)	0 (0–1)	0.5 (0.7)	0 (0–1)
Hernia site	Infiltration of trocar entry points	0.3 (0.5)	0 (0–1)	0.3 (0.5)	0 (0–1)	0.2 (0.4)	0 (0–1)
	Blockage of transverse abdominal muscle	0 (0)	0 (0–0)	0 (0)	0 (0–0)	0 (0)	0 (0–0)

*SD, standard deviation; IR, intra-quadratic range*.

#### Analgesia Method

The infiltration of trocar entry points with local analgesic agents was associated with greater pain scores at the trocar site compared to the TAP-block at 6, 12 and 24 h postoperatively (*p* < 0.001).

Regarding pain at hernia area, it was demonstrated that infiltration of trocar sites with local analgesics was associated with significantly higher pain scores compared to TAP-block at 6 and 12 h after surgery (*p* = 0.032), whereas pain scores at 24 h after surgery were not significantly higher for trocar site infiltration with local analgesics (*p* = 0.072).

#### Pain Area

Using infiltration of trocar entry sites with local analgesics, pain scores were significantly higher near the trocar insertion site at 6, 12 and 24 h postoperatively compared to the hernia area (*p* < 0.001).

The utilization of TAP-block was associated with higher pain scores at the trocar placement sites at 6, 12 and 24 h postoperatively, compared to the pain at hernia site (*p* = 0.006, *p* = 0.004 and *p* = 0.003, respectively).

#### Time Intervals

**Figure 2:**
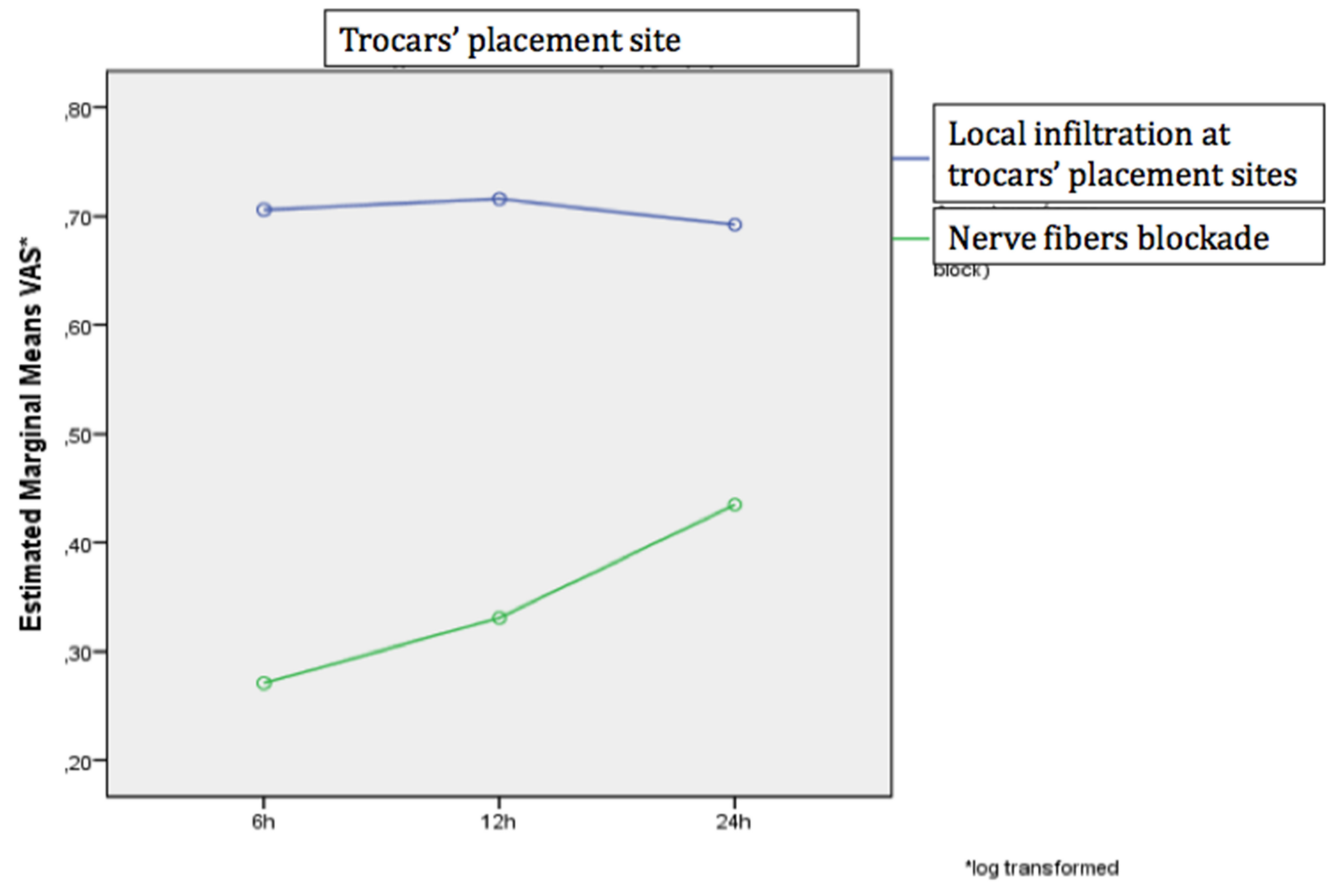
The change in the VAS scale over time separately for the two analgesic methods at the site of tool placement.

**Figure 3:**
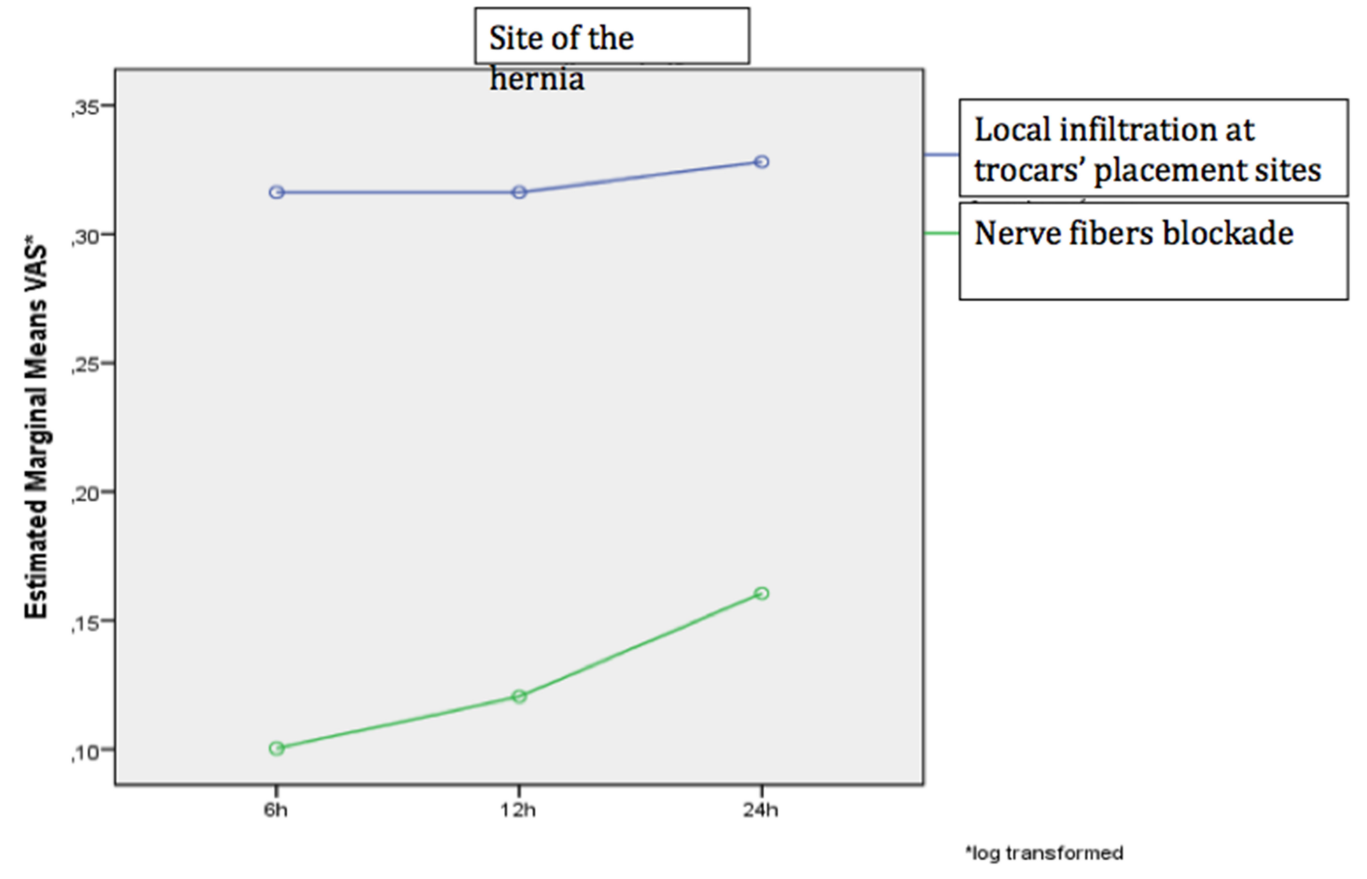
The change in the VAS scale over time separately for the two analgesic methods at the hernia site.

No significant differences were observed between pain scores at 6, 12 and 24 h after surgery using both methods of analgesia (local infiltration of trocar sites and TAP-block) or assessing pain at both areas (trocar insertion point and hernia area) *(p* > 0.05). In addition, the degree of pain alteration was similar regardless to the pain assessment site and the anesthetic method (*p* > 0.05) (Figures 2, 3).

### Pain During Movement

Pain scores during movement near the trocar insertion site and near the hernia area according to the 10-point VAS scale are presented in Table 3.

**Table 3:** Pain scores according to the 10-point VAS scale of patients during movement measured at the trocar and hernia site.

		**6 h**		**12 h**		**24 h**	
		**Mean (SD)**	**Median (range)**	**Mean (SD)**	**Median (range)**	**Mean (SD)**	**Median (range)**
Trocar placement site	Infiltration of trocar entry points	4.1 (0.7)	4 (4–5)	4.3 (0.9)	4 (4–5)	4 (0.9)	4 (3–5)
	Blockage of transverse abdominal muscle	1.3 (1.4)	1 (0–3)	1.5 (1.3)	1 (0–3)	1.9 (1)	2 (1–3)
Hernia site	Infiltration of trocar entry points	1.1 (0.5)	1 (1–1)	1.1 (0.5)	1 (1–1)	1.2 (0.6)	1 (1–2)
	Blockage of transverse abdominal muscle	0.3 (0.5)	0 (0–1)	0.4 (0.5)	0 (0–1)	0.5 (0.5)	1 (0–1)

*SD, standard deviation; IR, intra-quadratic range*.

#### Analgesia Method

The infiltration of trocar entry points with local analgesic agents was associated with greater pain scores at the trocar site compared to the TAP-block at 6, 12 and 24 h postoperatively (*p* < 0.001).

Regarding pain at hernia area, it was demonstrated that infiltration of trocar sites with local analgesics was associated with significantly higher pain scores compared to TAP-block at 6 and 12 h after surgery (*p* < 0.001), as well as at 24 h postoperatively (*p* = 0.003).

#### Pain Area

Using infiltration of trocar entry sites with local analgesics, pain scores were significantly higher near the trocar insertion site at 6, 12 and 24 h postoperatively compared to the hernia area (*p* < 0.001).

The utilization of TAP-block was associated with higher pain scores at the trocar placement sites at 6, 12 and 24 h postoperatively, compared to the pain at hernia site (*p* < 0.001).

#### Time Intervals

No significant differences were observed between pain scores at 6, 12 and 24 h after surgery using local infiltration of trocar sites at both pain assessment areas (trocar insertion point and hernia area) *(p* > 0.05). When TAP-block was utilized, no significant changes in pain scores over time at the hernia site was reported (*p* > 0.05). However, near the area of trocar insertion, pain scores at 24 h were significantly greater compared to 6 and 12 h after surgery (*p* < 0.001 and *p* = 0.002, respectively). In addition, pain scores at 12 h were significantly higher compared to 6 h after surgery (*p* < 0.05).

**Figure 4:**
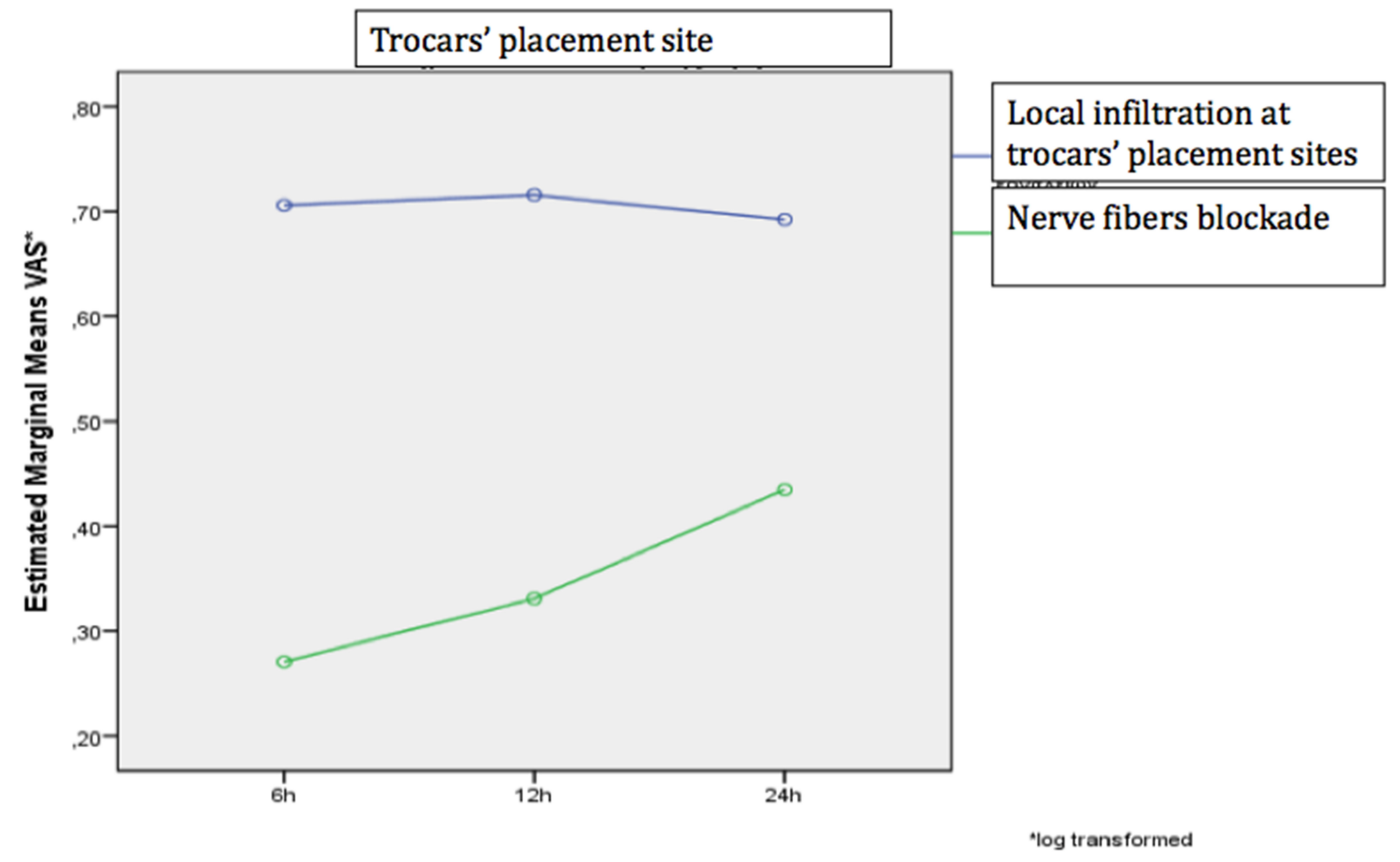
The change in the VAS scale over time separately for the two analgesic methods when performed at the site of tool placement.

**Figure 5:**
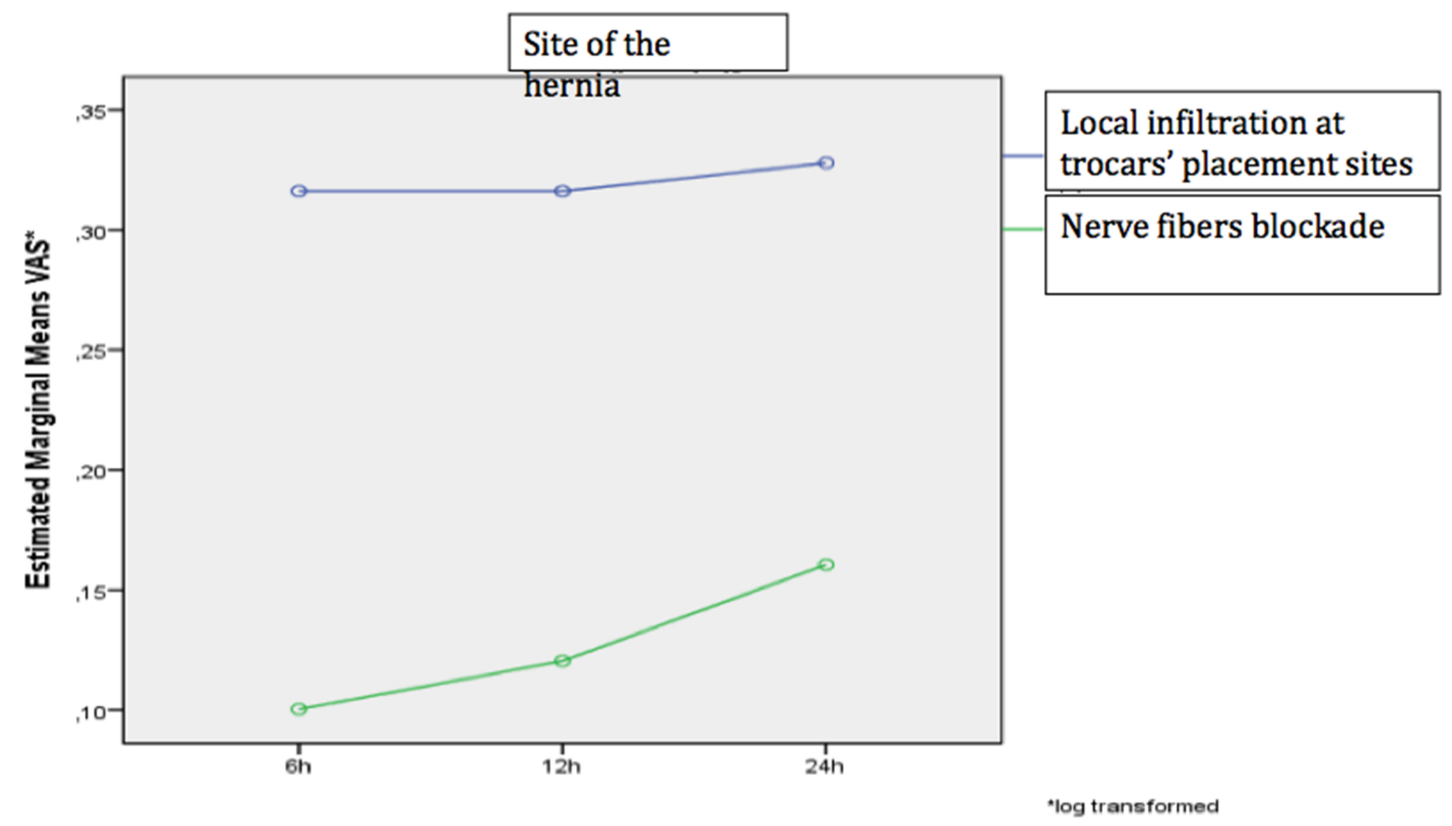
The change in the VAS pain numerical scale over time for the two analgesic methods when performed at the hernia site.

The degree of pain alteration varied significantly depending on the assessment site and analgesia method (*p* = 0.019), as a significant change was demonstrated when pain was assessed near the trocar insertion site and TAP-block was utilized (Figures 4, 5).

### Side Effects and Administration of an Additional Dose of Analgesia

No patient experienced any side effects from either local trocar site infiltration or TAP-block. Moreover, no patient needed any additional doses of analgesia.

## Discussion

### Postoperative Pain Management

The present study demonstrated that postoperative pain after laparoscopic groin hernia repair was significantly decreased in patients who underwent TAP-block instead of local anesthetic infiltration, especially at the trocar site. It is noteworthy that this area seemed to be the most painful after laparoscopic groin hernia repair, regardless of the analgesic technique applied. The hernia area accounted for less pain using both types of analgesia. In addition, intraoperative TAP-block under direct visualization seemed to be effective in relieving immediate postoperative pain, especially at the level of somatosensory pain, as the assessment of visceral pain was not included in the scopes of our study. Finally, it was reported that TAP-block was superior compared to the local anesthetic infiltration technique.

Similar results have been reported in previous studies (12, 13). Nevertheless, an important difference is that TAP-block was performed under US-guidance (14, 15), whereas in our study the nerve fiber blockade was performed intraoperatively under direct vision. The decreased visibility in patients with increased body mass index (BMI) due to significant amount of pre-peritoneal fat was the main difficulty that was encountered during intraoperative TAP-block under direct vision. On the other hand, complications of TAP-block are rare. In most cases they pertain to intra-abdominal injury (hematoma and liver injury), either when attempted by anatomical landmarks or even under US-guidance (16). An important advantage of nerve fiber blockade under direct vision is the prevention of intra-abdominal organ injury, although hematoma cannot be always avoided. Moreover, intraoperative blockade under direct vision requires less time, does not require additional equipment and decreases operational cost. According to a comparative study by Narasimhulu et al. (9), the time needed for the intraoperative technique was significantly shorter compared to the ultrasound technique (2.4 vs. 12.1 min, *p* < 0.001).

### Clinical Practice

The small number of patients who were recruited in the present study after retrospective search of the records of a high-volume university hospital demonstrate the limited familiarity with this technique. However, the promising results of our study could pave the way to a more systematic use of this technique. The future utilization of this technique in a large number of patients could highlight its effectiveness and potential difficulties that need to be addressed. In contrast to modern but expensive and scarcely available surgical options for groin hernia repair, such as robotic surgery, nerve blockade under direct vision offers an effective modification of laparoscopic groin hernia repair while enhancing trainee education and improving patients' outcomes (17). Furthermore, TAP-block under direct vision seems to decrease postoperative pain and increases quality of life, satisfaction and subsequently compliance of patients, which are goals that have been projected in the context of biopsychosocial clinical practice (18). Finally, the broad use of this technique promotes medical education as well. Learning analgesia techniques could be an important acquisition during specialization. Performing nerve blockade without ultrasound guidance facilitates learning for both trainers and trainees.

### Health Services Cost

Reduction of postoperative pain contributes to the faster recovery of patients and return to their daily activities. Data from the US demosntrate that post-operative pain still remains a serious problem for elderly patients even nowadays (19). Postoperative pain management by opioid medication reduces the mobility of the digestive system and the overall physical and mental activity of patients. Moreover, it predisposes to respiratory complications (atelectasis), cardiovascular complications (hypertension, myocardial ischemia, venous thrombosis) and predisposes to the development of chronic pain. All these lead to an increase of health expenses, both direct costs (extension of hospitalization, insurance reimbursement) and indirect costs (patient and caregivers salary, inability of the patient to return to his professional activity) (19). According to a recent study from the US, the direct cost of treating postoperative pain could increase the total cost of hospitalization by $647–$694 per patient. Furthermore, cost-benefit studies of gastrointestinal disorders have shown that indirect costs may be equal or even greater than direct costs (19).

The analgesic technique proposed in our study helps to reduce total health cost by relieving health system from additional cost of special equipment or training in ultrasound techniques. In addition, this technique promotes creating one-day surgery centers, which are expected to relieve surgery clinics and further reduce the cost of treatment. However, patient discharge after groin hernia repair must be determined by the principles of evidence-based medicine, rather than by the estimated cost (20).

### Limitations and Future Research

The present study includes a small sample of patients, that was a result of power statistical analysis, which is an important limitation. In addition, the lack of randomization and the retrospective nature of our investigation seem to be limitations as well. However, our study demonstrated that TAP-block under direct vision could be more effective compared to local analgesic infiltration in reducing postoperative pain at the trocar insertion site after laparoscopic groin hernia repair. To the best of our knowledge, it is the first pilot clinical study which demonstrates the effect of this technique in laparoscopic groin hernia repair. Therefore, a large, prospective, multicenter, doubly randomized study, with a homogeneous sample of patients, materials and techniques, could make these outcomes more robust. Allocating legs of such a study to the estimation of costs, patient satisfaction and educational outcomes would be useful as well.

## Conclusions

Intraoperative TAP-block under direct vision seems to be an effective, fast and easy technique in order to reduce postoperative pain after laparoscopic groin hernia repair instead of using local analgesic infiltration. In comparison to TAP-block under US-guidance, this technique seems to be superior in the terms of feasibility, speed and reproductivity. However, more studies are required to validate these results in a prospective and randomized context.

## Data Availability Statement

The raw data supporting the conclusions of this article will be made available by the authors, without undue reservation.

## Ethics Statement

The studies involving human participants were reviewed and approved by IRB - Hippocration General Hospital of Athens. Written informed consent to participate in this study was provided by the participants' legal guardian/next of kin.

## Author Contributions

AC: study design, data analysis, and writing. MF and GG: data collection and writing. EK: writing. TX: study design. NM: data analysis and writing. All authors contributed to the article and approved the submitted version.

## Funding

This work was supported by National and Kapodistrian University of Athens Special Account for Research Grants (UoA/S.A.R.G.).

## Conflict of Interest

The authors declare that the research was conducted in the absence of any commercial or financial relationships that could be construed as a potential conflict of interest.

## Publisher's Note

All claims expressed in this article are solely those of the authors and do not necessarily represent those of their affiliated organizations, or those of the publisher, the editors and the reviewers. Any product that may be evaluated in this article, or claim that may be made by its manufacturer, is not guaranteed or endorsed by the publisher.
